# Butein inhibits cell proliferation and induces cell cycle arrest in acute lymphoblastic leukemia via FOXO3a/p27kip1 pathway

**DOI:** 10.18632/oncotarget.7624

**Published:** 2016-02-23

**Authors:** Yan-Lai Tang, Li-Bin Huang, Wen-Hao Lin, Li-Na Wang, Yun Tian, Dingbo Shi, Jingshu Wang, Ge Qin, Anchuan Li, Yan-Ni Liang, Huan-Juan Zhou, Zhi-Yong Ke, Wenlin Huang, Wuguo Deng, Xue-Qun Luo

**Affiliations:** ^1^ Department of Pediatrics, The First Affiliated Hospital, Sun Yat-sen University, Guangzhou, China; ^2^ Sun Yat-sen University Cancer Center, State Key Laboratory of Oncology in South China, Collaborative Innovation Center of Cancer Medicine, Guangzhou, China; ^3^ Department of Radiation Oncology, Fujian Medical University Union Hospital, Fuzhou, China; ^4^ State Key Laboratory of Targeted Drug for Tumors of Guangdong Province, Guangzhou Double Bioproduct Inc., Guangzhou, China

**Keywords:** butein, acute lymphoblastic leukemia, FOXO3α, p27kip1

## Abstract

Acute lymphoblastic leukemia (ALL) is a common hematological malignancy characterized by the uncontrolled proliferation of leukemia cells in children. Discovering and developing effective chemotherapeutic drugs are needed for ALL. In this study, we investigated the anti-leukemic activity of butein and its action mechanisms in ALL. Butein was found to significantly suppress the cellular proliferation of ALL cell lines and primary ALL blasts in a dose-dependent manner. It also induced cell cycle arrest by decreasing the expression of cyclin E and CDK2. We also found that butein promoted nuclear Forkhead Class box O3a (FOXO3a) localization, enhanced the binding of FOXO3a on the p27kip1 gene promoter and then increased the expression of p27kip1. Moreover, we showed that FOXO3a knockdown significantly decreased the proliferation inhibition by butein, whereas overexpression of FOXO3a enhanced the butein-mediated proliferation inhibition. However, overexpression of FOXO3a mutation (C-terminally truncated FOXO3a DNA-binding domain) decreased the proliferation inhibition by butein through decreasing the expression of p27kip1. Our results therefore demonstrate the therapeutic potential of butein for ALL via FOXO3a/p27kip1 pathway.

## INTRODUCTION

Although the dramatic improvements in disease-free survival among children with acute lymphoblastic leukemia (ALL) has been achieved, outcomes for those who fail to achieve long-term disease-free survival or relapse are poor [1, 2]. Given that the current chemotherapy regimens have had limited success in improving survival in refractory or relapsed ALL, the efforts to investigate potential application of natural anti-leukemic agents are necessary [3, 4].

Natural products that existing in plants, such as vincristine, have been shown to play a major role in anti-leukemic effect [5]. Butein, 3,4,2′,4′-Tetrahydroxychalcone, is a polyphenolic compound that has been identified from a number of plants, such as *Caragana jubata* and *Rhus verniciflua* [6]. Recently, butein has been shown to exhibit anti-tumor activity against breast cancer [7], bladder cancer [8], prostate cancer [9] and mesothelioma [10]. Butein has inhibited CXCR4 expression, which is correlated with the inhibition of CXCL12-induced migration and invasion in breast and pancreatic cancer cells [11]. In addition, butein has been found to suppress proliferation, induce apoptosis and overcome gefitinib-resistance in lung cancer via EGFR/MET signaling pathway [12]. Moreover, butein has inhibited the growth of xenografted human colorectal tumors and hepatocellular carcinoma in vivo [13, 14]. In addition to solid tumors, butein has been proved to inhibit telomerase activity and proliferation, induce apoptosis and differentiation in leukemia cells through Akt/hTERT pathway [15]. Furthermore, butein could reverse the TRAIL-resistance of human myeloid leukemia U937 cells [16]. Although it has been shown that butein could suppress proliferation, induce apoptosis and differentiation in myeloid leukemia cells, its molecular mechanisms responsible for inhibition of cell growth and cell cycle progression in acute lymphoblastic leukemia are yet unknown.

In this study, we investigated the effect of butein on cellular proliferation and cell cycle arrest in ALL cell lines and primary leukemic blasts from pediatric ALL. Additionally, we also identify the role of butein in the regulation of the nuclear translocation of Forkhead Class box O3a (FOXO3a) and the p27kip1 signaling pathway in ALL cells. Our results indicate that butein would serve as a potential candidate targeting FOXO3a to promote p27kip1 expression for anti-leukemic treatment.

## RESULTS

### Butein inhibits the proliferation of ALL cells in a dose-dependent manner

**Figure 1:**
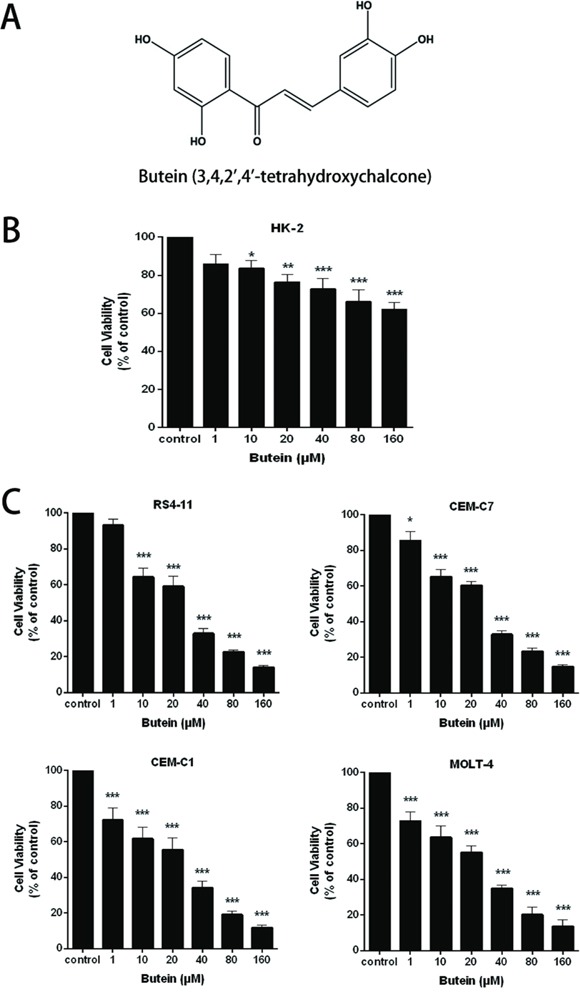
Butein inhibited the proliferation of ALL cells **A.** The molecular structure of butein. **B.** HK-2 cell line was treated with 0, 1.0, 10, 20, 40, 80 or 160 μM butein for 24 h. The cell proliferation was tested by the MTS assay. **C.** RS4-11, CEM-C7, CEM-C1 and MOLT-4 cells were exposed to different concentrations of butein for 24 h. The cell proliferation was tested by the MTS assay.*P < 0.05, **P < 0.01 and ^***^P < 0.001 vs control. The results are presented as the mean± s.d. of three separate experiments.

The molecular structure of butein was showed in Figure 1A. To evaluate the effects of butein on the renal toxicity of human normal proximal tubular cell and the proliferation of ALL cells, we examined the viability of HK-2 cell line and ALL cells. As shown in Figure 1B and Figure 1C, various concentrations of butein remarkably inhibited the proliferation of the ALL cell lines (RS4-11, CEM-C7, CEM-C1 and MOLT-4) in a concentration-dependent manner. Compared to ALL cell lines, different concentrations of butein didn't remarkably inhibit the viability of HK-2 cell.

### Butein suppresses the viability of ALL cells at different treatment times

**Figure 2:**
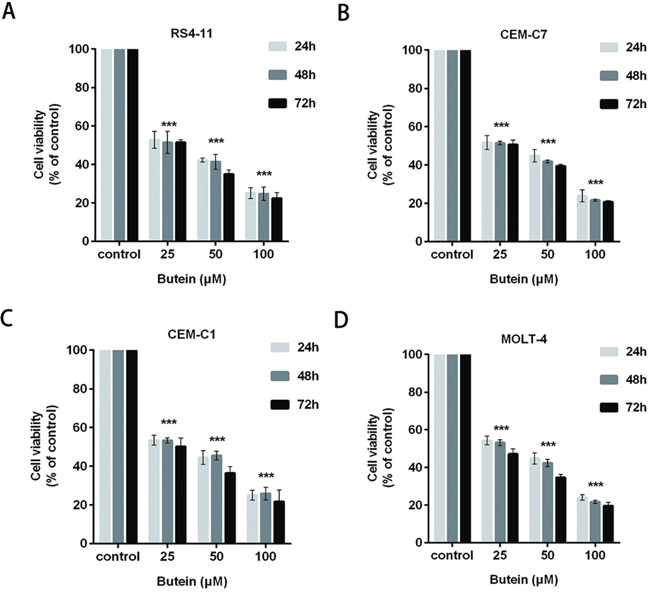
Butein inhibited the viability of ALL cells at different times The cell viability was examined using MTS assay in four ALL cell lines RS4-11 **A.** CEM-C7 **B.** CEM-C1 **C.** and MOLT-4 **D.** exposed to 25, 50 or 100 μM butein for 24 h, 48 h and 72 h. ^***^P < 0.001 vs control. The results are presented as the mean± s.d. of three independent experiments.

We also tested theinhibition of proliferation of ALL cells exposed to 0, 25, 50 or 100 μM butein for 24, 48, and 72 h. Butein significantly inhibited the viability and proliferation of RS4-11 (Figure 2A), CEM-C7 (Figure 2B), CEM-C1 (Figure 2C) and MOLT-4 (Figure 2D) cell lines at different treatment times.

### Butein inhibits the growth of primary ALL cells ex vivo

**Figure 3:**
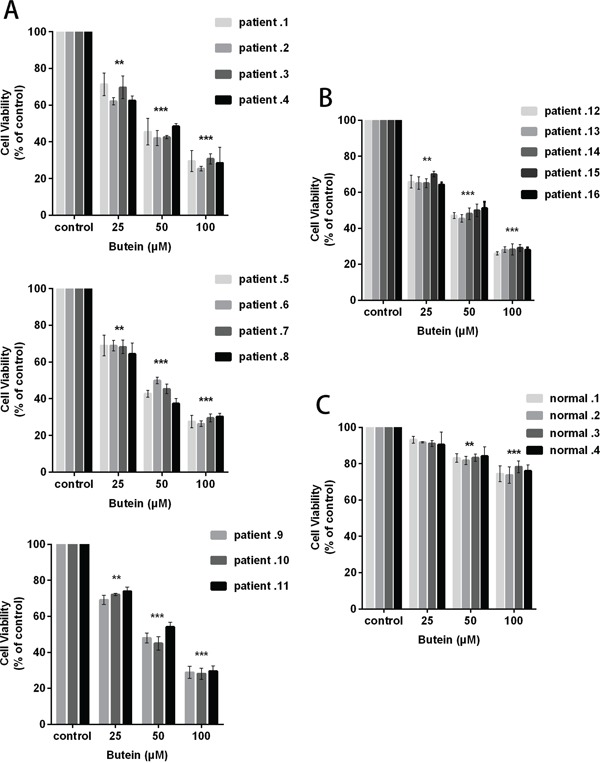
Butein inhibited the proliferation of primary ALL blasts ex vivo 11 B-ALL primary cells **A.** 5 T-ALL primary cells **B.** and 4 normal mononuclear cells **C.** were exposed ex vivo to 25, 50 or 100 μM butein for 24 h. Cell viability was measured using MTS assay. **P < 0.01 and ^***^P < 0.001 vs control. The results represent the means±s.d. of triplicates.

To examine the effect of butein on primary B-ALL blasts, T-ALL blasts and normal mononuclear cells, we analyzed the cell proliferation using the MTS assay. We exposed these cells to 0, 25, 50, or 100 μM butein for 24 h. As shown in Figure 3A, the growth of B-ALL blasts was markedly inhibited in a dose-dependent manner. The similar result was obtained in T-ALL blasts (Figure 3B). Interestingly, treatment with butein resulted in the dose-dependent growth inhibition of primary ALL cells, but has no cytotoxicity in normal mononuclear cells at the same dose (Figure 3C).

### Butein induces cell cycle arrest in ALL cells

**Figure 4:**
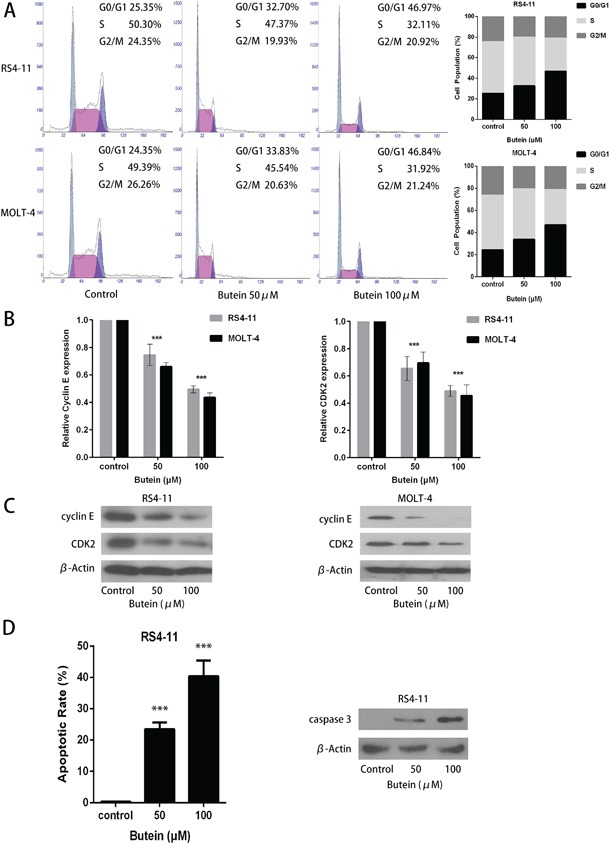
Butein induced cell cycle arrest and inhibited the activation of CDK2-Cyclin E complex in RS4-11 and MOLT-4 cells **A.** Cells were treated with 0, 50 or 100 μM butein for 24 h. Flow cytometric analysis was used to determine cell cycle distribution of RS4-11 and MOLT-4 cells. **B.** The expression levels of cyclin E and CDK2 mRNAs in RS4-11 and MOLT-4 cells were detected by qRT-PCR analysis. **C.** Western blot detected the protein levels of cyclin E and CDK2 in the indicated cells exposed to 0, 50 or 100 μM butein. **D.** Flow cytometric analysis was used to determine cell apoptosis of RS4-11 cell, western blot detected the protein levels of caspase 3 in RS4-11 cell exposed to 0, 50 or 100 μM butein. ^***^P < 0.001 vs control. The results are presented as the mean± s.d. of three separate experiments.

We also analyzed the effects of butein on cell cycle of ALL cells. As shown in Figure 4A, treatment with 0, 50 or 100 μM butein resulted in accumulation of RS4-11 and MOLT-4 cells in S-phase. To explore the underlying molecular mechanism of cell cycle arrest induced by butein in ALL cells, we detected the effect of butein on the expression of cell cycle proteins. QRT-PCR analysis showed a significant down-regulation of cyclin E and CDK2 in the butein-treated groups in RS4-11 and MOLT-4 cells (Figure 4B). Western blot analysis also showed that butein treatment caused the dose-dependent decreases of cyclin E and CDK2 proteins in RS4-11 and MOLT-4 cell lines (Figure 4C). In addition, compared with the control group, butein treatment resulted in a significant increase in apoptotic rates and the caspase 3 expression in RS4-11 cell line (Figure 4D).

### Butein activates FOXO3a/p27kip1 pathway in ALL cell lines

**Figure 5:**
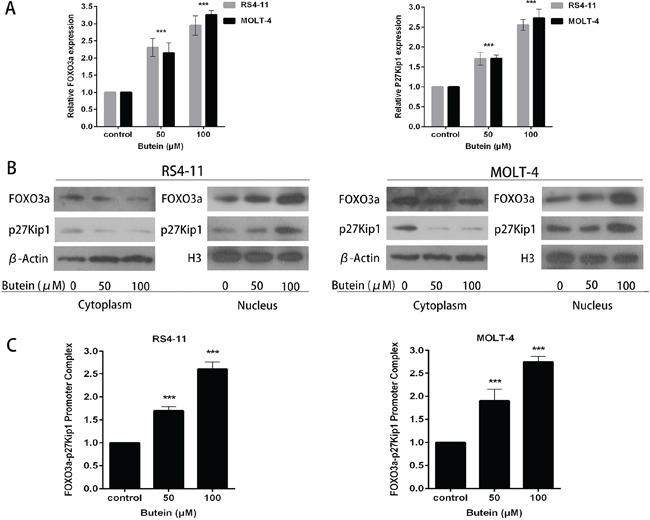
Butein induced the activation of the FOXO3a/p27kip1 pathway **A.** The cells were treated with 0, 50 or 100 μM butein. The expression of FOXO3a and p27kip1 mRNA were assessed using qRT-PCR. **B.** Cells were exposed to 0, 50 or 100 μM butein for 24 h, and then cytoplasm and nucleus extracts were prepared. Equal amounts of protein from each fraction were analyzed by immunoblotting with anti-FOXO3a and anti-p27kip1. β-actin was used as an internal control for the cytoplasm fraction and H3 for the nucleus fraction. **C.** Quantitative ChIP analysis of FOXO3a binding to p27kip1 promoter sequences in the indicated cells exposed to 0, 50 or 100 μM butein. ^***^P < 0.001 vs control. The results are presented as the mean± s.d. of at least three independent experiments.

The FOXO3a expression has been demonstrated to inhibit cell proliferation and induce cell cycle arrest in glioma cells [17]. To determine whether butein could affect FOXO3a signaling pathway in ALL cells, the expression levels of FOXO3a and p27kip1, one of FOXO3a downstream target genes, were detected by qRT-PCR and Western blot. As shown in Figure 5A, butein effectively increased the expression of FOXO3a and p27kip1 at mRNA level in RS4-11 and MOLT-4 cells in a dose-dependent manner. Similarly, we found that butein also effectively promoted FOXO3a and p27kip1 expression at protein level in ALL cell nucleus (Figure 5B).

We further evaluated the effect of butein on the binding activity of FOXO3a on p27kip1 promoter by ChIP assay. The results showed that treatment of cells with butein markedly promoted the binding of FOXO3a on the p27kip1 promoter in chromatin structure in a dose-dependent manner as compared with the control treatment (Figure 5C).

### FOXO3a knockdown attenuated the anti-proliferation activity of butein

**Figure 6:**
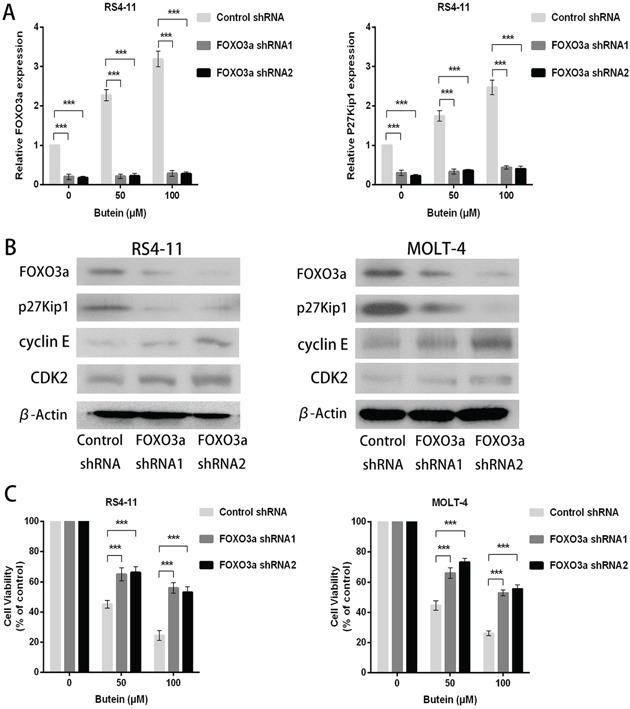
FOXO3a knockdown promoted cellular proliferation in ALL cells treated with different concentrations of butein **A.** qRT-PCR was used to detect the mRNA levels of FOXO3a and p27kip1 after FOXO3a shRNA knockdown in the indicated cells exposed to 0, 50 or 100 μM butein. **B.** Western blot analysis was used to analyze the cyclin E, CDK2, FOXO3a and p27kip1 protein expression in ALL cells with FOXO3a shRNA knockdown. **C.** The cell viability was examined using MTS assay in control and FOXO3a knockdown cells treated with 0, 50 or 100 μM butein. ^***^P < 0.001 vs control. The results represent the means±s.d. of triplicates.

To further elucidate the mechanisms of the dose-dependent increase of FOXO3a and p27kip1 expression in ALL cells treated with butein, we constructed lentiviral shRNA of FOXO3a in RS4-11 and MOLT-4 cells. FOXO3a knockdown with two different shRNA (shRNA1 and shRNA2) inhibited FOXO3a and p27kip1 mRNA levels in ALL cells treated with different concentrations of butein (Figure 6A). We also found that FOXO3a and p27kip1 protein expression were both inhibited and the protein expression of cyclin E and CDK2 were increased by FOXO3a knockdown (Figure 6B). FOXO3a knockdown attenuated the proliferation inhibition mediated by butein at different concentrations (Figure 6C).

### FOXO3a overexpression promotes the anti-proliferation activity of butein

**Figure 7:**
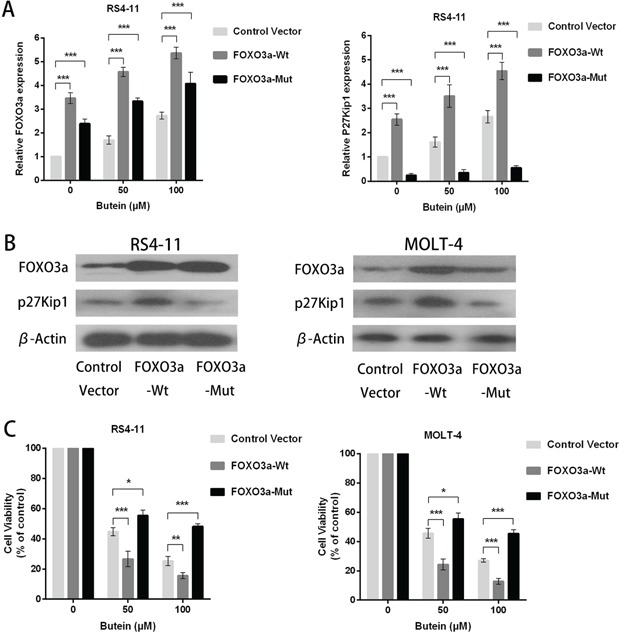
FOXO3a overexpression enhanced the anti-proliferation activity of butein in ALL cells treated with different concentrations of butein **A.** QRT-PCR analysis of FOXO3a and p27kip1 mRNA after FOXO3a overexpression or FOXO3a mutation (C-terminally truncated FOXO3a DNA-binding domain) in RS4-11 and MOLT-4 cells exposed to 0, 50 or 100 μM butein. **B.** Western blot analysis of FOXO3a and p27kip1 proteins after FOXO3a overexpression or FOXO3a mutation. **C.** The cell viability was tested using MTS assay in control and FOXO3a overexpression or FOXO3a mutation cells treated with 0, 50 or 100 μM butein. *P < 0.05 and ^***^P < 0.001 vs control. The results are presented as the mean± s.d. of at least three independent experiments.

To further confirm the action mechanisms of butein, we also constructed lentiviral wild-type FOXO3a and mutant FOXO3a (C-terminally truncated FOXO3a DNA-binding domain) in RS4-11 and MOLT-4 cells. FOXO3a overexpression increased FOXO3a and p27kip1 mRNA levels, but FOXO3a mutation decreased p27kip1 mRNA levels (Figure 7A). The expression of FOXO3a and p27kip1 proteins were also increased by FOXO3a overexpression, but FOXO3a mutation decreased p27kip1 protein expression (Figure 7B). FOXO3a overexpression promoted the proliferation inhibition by butein at different concentrations, but FOXO3a mutation attenuated the butein-mediated inhibition of proliferation of ALL cells (Figure 7C).

## DISCUSSION

Acute lymphoblastic leukemia (ALL) is the most common pediatric cancer, and modern treatment strategies have led to progressive improvements in survival [4, 18]. However, the treatment of refractory or relapsed ALL is still challenging [19], whereas prognosis of relapsed pediatric ALL depends primarily on the time of relapse after initial therapy, sites of relapse, and immunophenotype [3, 20]. Efforts to discover several innovative therapies using novel targeted treatment are necessary [21].

Our previous study revealed that flavokawain B, one of the flavokawains extracted from kava, exhibited anti-leukemic activity through the activation of the p53 and caspase-dependent pathways in ALL, it may be a promising agent for the treatment of patients with ALL [22]. Based on previous reports suggesting that butein, a bioactive flavonoid extracted from numerous native plants, could inhibit proliferation and induce apoptosis in numerous human cancer cells [23–27], we analyzed the anti-leukemic effect of butein on ALL cells. In the present study, we found that butein effectively inhibited ALL cells growth with IC50 of about 20 μM, while butein nearly had no adverse effect on human normal proximal tubular cells. Unlimitedness of cell cycle progress is a major cause in proliferation and neoplastic transformation [28]. Therefore, inducing cell cycle arrest could provide new opportunities for therapeutic strategies [29]. Our data showed that butein arrested the cell cycle at the G1/S transition following 24 hours treatment by decreasing the expression of cyclin E and CDK2.

The research on the molecular mechanisms of butein had focused on PI3K/AKT/mTOR pathway [23]. Targeting the PI3K/AKT/mTOR signaling pathway, a central signaling center of cellular growth and survival, may have pro-apoptotic and anti-proliferative effects on hematological malignancies [30, 31]. Recent studies have shown that combinations of mTOR inhibitors with other drugs enhanced anti-leukemic activities in acute myeloid leukemia (AML) cells [32–34]. The mTOR inhibitor BEZ235 overcame glucocorticoid resistance in pediatric T-cell ALL (T-ALL) by increasing BIM expression [35]. In addition to mTOR pathway, the PI3K/AKT signaling also targeted the FOXO3a/p27kip1 pathway to suppress proliferation and induce cell cycle arrest [36]. Our data showed that butein activated FOXO3a/p27kip1 pathway, the important downstream signaling of PI3K/AKT pathway, to suppress proliferation and induce cell cycle arrest in ALL. Targeting the PI3K/AKT/FOXO3a/p27kip1 pathway and PI3K/AKT/mTOR pathway may have synergistic effects on ALL. The combinations of butein with mTOR inhibitors may enhance anti-leukemic activities in ALL cells.

Recent reports showed that FOXO3a acted as a transcription factor that inhibited proliferation and cell-cycle progression at the G1/S transition by controlling transcription of the cyclin-dependent kinase inhibitor p27kip1 [17, 37–39]. In our study, we showed that butein promoted nuclear localization of FOXO3a, enhanced the binding ability between FOXO3a and the promoter of p27kip1 and then increased the expression of p27kip1, leading to inhibit proliferation and induce cell-cycle arrest at the G1/S transition. Furthermore, we showed that FOXO3a knockdown suppressed the proliferation inhibited by butein and overexpression of FOXO3a promoted cellular proliferation inhibited by butein, but FOXO3a mutation (C-terminally truncated FOXO3a DNA-binding domain) blocked the proliferation inhibited by butein. The recent study revealed that FOXO3a DNA-binding domain (FOXO3a-DBD), a FOXO consensus DNA-binding site, played an important role in promoter recognition by FOXO proteins and stabilizing the formation of the complex with DNA [40]. Our data revealed that the FOXO3a mutation lacking the C-terminal DNA-binding domain lost the ability to bind the promoter of p27kip1, leading to decrease the expression of p27kip1. It indicated that the C-terminal region of FOXO3a-DBD contributed significantly to the formation of a stable FOXO3a–p27kip1 complex.

**Figure 8:**
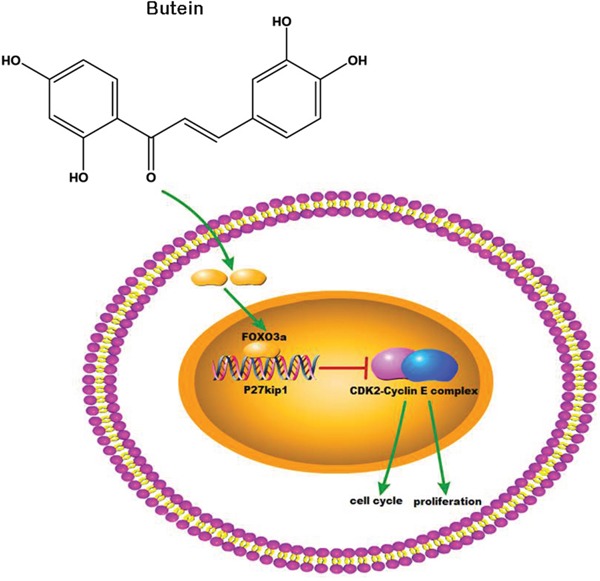
Butein activated the FOXO3a/p27kip1 signaling pathway in ALL Butein inhibited cellular proliferation and induced cell cycle arrest through activating the FOXO3a/p27kip1 signaling pathway and inhibiting the activation of CDK2-Cyclin E complex.

In conclusion, our study showed that butein inhibited cellular proliferation and induced cell cycle arrest in ALL cells via activating the FOXO3a/p27kip1 pathway. Butein increased the expression of FOXO3a, enhanced the binding ability between FOXO3a and p27kip1, and promoted the expression of p27kip1 (Figure 8). Additionally, butein significantly suppressed the growth of primary ALL. The data suggested that butein, used alone or in combination with other drugs, may be a promising drug for ALL treatment.

## MATERIALS AND METHODS

### Cell lines and cell culture

HK-2, HEK293T, RS4-11 (B-ALL), CEM-C7 (T-ALL), CEM-C1 (T-ALL), and MOLT-4 (T-ALL) cell lines were obtained from the American Type Culture Collection (ATCC, Manassas, VA, USA). The information of cell lines is detailed in Table 1. The ALL cells were cultured in RPMI 1640 medium (Invitrogen, Carlsbad, CA, USA) supplemented with 10% fetal bovine serum (HyClone, Logan, UT, USA). HK-2 and HEK293T were cultured in DMEM (Invitrogen, Carlsbad, CA, USA) containing 10% fetal bovine serum. All cells were cultured in a humidified atmosphere with 5% CO_2_ at 37°C.

**Table 1:** The characteristics of the cell lines and their IC50 values of buetin

Characteristics	Cell Lines				
	RS4-11	CEM-C7	CEM-C1	MOLT-4	HK-2
Cell type	B-ALL	T-ALL	T-ALL	T-ALL	PTC
Primary site	BM	PB	PB	PB	Kidney
IC50 (μM)	22.29	22.89	19.26	20.10	156.90

ALL, acute lymphoblastic leukemia; PTC, proximal tubular cell; BM, bone marrow; PB, peripheral blood; IC50, half-inhibitory concentration, calculated using Graphpad Prism 6 software with three independent values from MTS assays.

### Primary ALL samples

This study enrolled 5 T-ALL, 11 B-ALL patients and 4 normal volunteers from the First Affiliated Hospital of Sun Yat-sen University. Clinical information about the patients is detailed in Table 2. Informed consent was obtained from all patients, and the study was approved by the Ethics Committee of the First Affiliated Hospital of Sun Yat-sen University. All analyzed samples were collected at the time of diagnosis before treatment. Mononuclear cells from bone marrow specimens of patients were separated using Ficoll–Paque PREMIUM (GE Healthcare, Uppsala, Sweden). Samples that included more than 80% leukemic blasts were frozen and stored in liquid nitrogen before use.

**Table 2:** Clinical and biological features for patients in the study

Patient	Sex	Age (years)	WBC (10^9^/L)	FAB category	Fusion gene	Blast cells (%)
1	F	7	47.15	L2, BII	TEL/AML1	83.00
2	F	5	34.20	L1, BIII	BCR/ABL	85.70
3	M	3	54.35	L2, BIV	None	90.80
4	F	5	12.50	L1, BIII	TEL/AML1	84.60
5	M	4	10.56	L1, BII	None	83.50
6	M	5	12.20	L2, BIII	None	84.60
7	M	7	12.15	L3, BIII	BCR/ABL	85.80
8	F	11	30.53	L2, BIV	TEL/AML1	83.70
9	F	4	20.50	L3, BIII	None	84.90
10	F	3	10.32	L1, BII	None	83.60
11	M	9	8.90	L2, BIV	BCR/ABL	82.80
12	M	5	15.56	L2, T	SIL/TAL1	82.70
13	M	3	58.52	L3, T	None	90.50
14	F	2	85.60	L1, T	MLL/AF9	84.80
15	M	5	67.40	L2, T	None	85.00
16	F	3	12.85	L2, T	None	87.00

F, female; M, male; WBC, white blood cells; FAB, French – American – British.

### Lentivirus infection

The FOXO3a short-hairpin RNA (shRNA)-expressing constructs, the wild-type FOXO3a expressing vector and the mutant FOXO3a expressing vector (C-terminally truncated FOXO3a DNA-binding domain) were purchased from Clontech (Mountain View, CA, USA). The pLP1, pLP2 and pLP/VSVG packaging plasmids were purchased from Invitrogen (Carlsbad, CA, USA). The constructs were transfected into HEK293T cells along with the packaging plasmids, and the lentivirus-containing supernatants were used to transduce RS4-11 and MOLT-4 cells. Puromycin selection to establish stable cells began 24 h after virus infection.

### Reagents and antibodies

Butein (Merck Millipore, Billerica, MA, USA) was dissolved in dimethyl sulfoxide (Sigma, St Louis, MO, USA). The final concentration of dimethyl sulfoxide in the culture media was 0.01%. The solutions were stored at −20°C before use. The primary antibodies used in this study were as follows: anti-cyclin E (4129S) and anti-CDK2 (2546P) were from Cell Signaling Technology (Boston, MA, USA); anti-caspase 3 (ab32351), anti-FOXO3a (ab12162) and anti-p27kip1 (ab32034) were from Abcam (Cambridge, MA, USA); anti-β-actin (A1978) and anti-H3 (H9289) were from Sigma-Aldrich (St Louis, MO, USA).

### MTS assay

The cells were plated in 96-well plates at a concentration of 1 × 10^4^ cells/well and treated with the indicated concentrations of butein. After 24, 48, and 72 h incubation, cell viability was tested using the MTS assay (Promega, Madison, WI, USA) according to the manufacturer's instructions. All experiments were repeated three times.

### Cell cycle analysis

The Cell cycle was determined using the Cell Cycle Detection Kit (Nanjing Keygen, Nanjing, China). Cells were seeded at 1×10^5^ cells/ml in 6-well plates and treated with various concentrations of butein. After 24 h incubation, 1×10^5^ cells were harvested and washed twice with cold PBS and fixed with frozen 70% ethanol in PBS at 4°C overnight. Then, the suspension was stained with DNA-staining solution (3.4 mmol/L Tris-Cl (pH 7.4), propidium iodide (PI), 0.1% Triton X-100 buffer, and 100 mg/mL RNase A). Stained cells were determined by flow cytometry (Cytomics FC500 Flow Cytometer, Beckman Coulter) in each phase of the cell cycle.

### Apoptosis assay

The apoptotic rate was determined using the Annexin V-FITC Apoptosis Detection Kit (Nanjing Keygen, Nanjing, China) according to the manufacturer's protocol. The rate of apoptosis was determined using flow cytometry (Cytomics FC500 Flow Cytometer, Beckman Coulter).

### RNA extraction and quantitative real-time reverse transcription PCR (qRT-PCR)

Total RNA was isolated using the TRIzol reagent (Invitrogen, Carlsbad, CA, USA) and was reverse transcribed using a PrimeScript First Strand cDNA Synthesis Kit (TaKaRa, Tokyo, Japan) according to the manufacturer's instructions. The Q-PCR reaction was performed following the kit protocol, and amplification was performed using the Mx3005P Real-Time PCR System (Agilent, CA, USA). The relative mRNA expression was normalized to β-actin RNA levels and analyzed using the 2^−ΔΔCT^ method. The primers were synthesized by Invitrogen (Carlsbad, CA, USA). Sequences of primers (forward and reverse, respectively) were as follows: p27kip1: 5′-GTCAAACGTAAACAGCTCGAAT-3′ and 5′-TGCATAATGCTACATCCAACG-3′; β-actin: 5′-GGCACCCAGCACAATGAA-3′ and 5′-TAGAAGCATTTGCGGTGG-3′; FOXO3a: 5′-AGGGAAGTTTGGTCAATCAGAA-3′ and 5′-TGGAGATGAGGGAATCAAAGTT-3′; CDK2: 5′-CCAGGAGTTACTTCTATGCCTGA-3′ and 5′-AATCCGCTTGTTAGGGTCGTA-3′; Cyclin E: 5′-AGAAATGGCCAAAATCGACA-3′ and 5′-CCCGGTCATCATCTTCTTTG-3′.

### Chromatin immunoprecipitation assay

Chromatin immunoprecipitation was performed using the ChIP-IT^®^ Express Enzymatic Kit (Active Motif, Carlsbad, CA, USA) according to the manufacturer's protocol. Transcription factor binding was finally assessed by qRT-PCR using the following primers: p27kip1 promoter, forward, 5′-GTCCCTTCCAGCTGTCACAT-3′; reverse 5′-GGAAACCAACCTTCCGTTCT-3′.

### Western blot assay

After cells plated in 100-mm dishes with different concentrations of butein for 24 h, they were harvested, washed and lysed. Whole cell lysates were prepared by using Complete Lysis-M Reagent Kit (Roche, Basel, Switzerland). Nuclear–cytoplasmic fractionation was conducted using the NE-PER Nuclear and Cytoplasmic Extraction Reagents kit (Pierce, Illinois, USA) according to the manufacturer's protocol. Proteins were transferred to polyvinylidene difluoride membranes. The protein bands were visualized using the SuperSignal Chemiluminescent Substrates from Thermo Fisher Scientific.

### Statistical analysis

Statistical analysis was performed using SPSS17.0 software (Chicago, IL, USA). All quantitative data were expressed as the mean ± standard deviation (SD) from at least three independent experiments. All tests were two-sided. Differences were considered statistically significant at *P < 0.05, **P < 0.01, and ^***^P < 0.001.
